# Factors associated with maternal attention and distraction during breastfeeding and childcare: A cross-sectional study in the west of Iran

**DOI:** 10.1515/med-2025-1213

**Published:** 2025-06-09

**Authors:** Roya Ahmadiniyatabesh, Erfan Ayubi, Ensiyeh Jenabi, Elham Fatholahi, Seyedeh Zahra Masoumi, Neda Skini

**Affiliations:** Student Research Center, Hamadan University of Medical Sciences, Hamadan, Iran; Cancer Research Center, Institute of Cancer, Avicenna Health Research Institute, Hamadan University of Medical Sciences, Hamadan, Iran; Mother and Child Care Research Center, Hamadan University of Medical Sciences, Hamadan, Iran; Hamadan University of Medical Sciences, Hamadan, Iran

**Keywords:** breastfeeding, smartphones, attention, distraction, mother, cross-sectional study

## Abstract

**Background:**

This study was designed to address factors associated with maternal attention and distraction during breastfeeding and childcare and the role of smartphones in western Iran.

**Methods:**

This cross-sectional study was conducted among 280 breastfeeding mothers in Iran in 2024. The data collection tools included a demographic-obstetric questionnaire and the Maternal Distraction Questionnaire. Data were analyzed using SPSS software, version 16, and the significance level was set less than 0.05.

**Results:**

The highest distraction during breastfeeding was associated with mobile phone use (62.14%), while the lowest was linked to reading (6.78%.). Mobile phone use was the highest distraction during breastfeeding and baby care (79.64%), while the lowest was reading a book (12.86%). Additionally, individual and social variables, such as education level, number of children, and economic status, were significantly associated with maternal attention and distraction levels (*p* < 0.05).

**Conclusion:**

Distraction during breastfeeding and during child care between women with three or more children was higher compared with women with one child. Additionally, distraction during child care between women with three or more children was higher compared with women with two children.

## 1 Introduction

Breast milk is recognized as a valuable resource with numerous positive effects on the growth and development of children [1]. Renowned global health organizations, such as the American Academy of Pediatrics, recommend exclusive breastfeeding for the first six months of an infant’s life [2]. Today, global attention is increasingly focused on the importance of early life as a golden opportunity to improve child health [3]. Despite these recommendations, the global rate of achieving exclusive breastfeeding remains low, with only 41% of infants being exclusively breastfed until six months of age [4]. Breastfeeding is considered one of the most effective strategies for improving child health in various communities [5]. However, numerous factors can affect breastfeeding. Despite the potential benefits of using media technologies, the use of these technologies has been recognized as a distraction that may impact the breastfeeding experience [6,7]. Research indicates that these modern technologies might negatively influence the quality of breastfeeding and, consequently, child health outcomes [8]. The findings of Arezi et al. [9], which investigated the impact of technology and digital media, underscore the importance of understanding and managing factors contributing to maternal distraction during breastfeeding and childcare. They reported that young mothers with lower levels of education experience significantly high levels of distraction while caring for their babies or breastfeeding [9]. Additionally, a study by Ventura et al. [10] revealed a link between maternal distraction during breastfeeding and reduced attention to the infant’s needs. The growing use of digital technologies, particularly mobile phones, has been identified as a significant source of distraction during breastfeeding [11]. The study by Golen and Ventura found that mothers who use their mobile phones more frequently are more prone to distractions [12].

Given the increasing use of digital technologies and the influence of modern environments on daily life, identifying and analyzing factors contributing to maternal attention and distraction during breastfeeding and childcare. Therefore, this study was designed to address factors associated with maternal attention and distraction during breastfeeding and childcare and the role of smartphones in western Iran.

## 2 Materials and methods

### 2.1 Study setting and study design

This cross-sectional study was conducted among breastfeeding mothers referred to Comprehensive Health Service Centers in Hamadan, Iran, between August and November 2024. Women who exclusively breastfed were invited to participate in the study.

### 2.2 Inclusion and exclusion criteria

The inclusion criteria for the study were: mother’s age over 18 years, between 2 weeks and 6 months since childbirth, baby’s age ≤6 months, exclusive breastfeeding, full-term and healthy singleton infants, and no breast abnormalities. Participants with incomplete questionnaires were excluded from the study.

### 2.3 Sampling method

A multi-stage sampling method was used. Hamadan City has 25 comprehensive health service centers; 6 centers were selected from different areas of Hamadan based on geographical location, and participants were then randomly selected from the clients of these centers. These six centers from different geographical regions were included in the study, representing a range of socioeconomic statuses, educational levels, and age groups. This diversity helps to reduce selection bias. Questionnaires were completed by those who agreed to participate.

### 2.4 Sample size

The effect size *f* from the analysis of variance (ANOVA) test was calculated based on the mean distraction scores across the study groups to estimate the sample size. An effect size *f* of 0.25 was selected, indicating a medium effect size for comparing scores across a variable with four levels. With a Type I error probability of 0.05 and a power of 90%, the required sample size was determined to be 232 participants. To account for a 20% margin for non-response and missing data, the final sample size was set at 280 participants.

### 2.5 Measurement

The data collection tools included a demographic-obstetric questionnaire and the Maternal Distraction Questionnaire (MDQ).

#### 2.5.1 Demographic-obstetric questionnaire

The demographic-obstetric questionnaire comprised the demographic section, which covered age, education level, occupation, family income status (mother’s perception of family economic status), baby’s gender, number of children, and type of delivery.

#### 2.5.2 MDQ

The MDQ was developed by Ventura et al. [13] to assess maternal distraction through self-report and includes various activities that mothers may engage in while interacting with their infants. This questionnaire contains 14 items. The first section consists of 10 questions about the frequency of common activities, such as watching TV, talking or texting on the phone, using the computer, or reading a magazine, that mothers may engage in during feeding or caregiving. Each question is rated on a 5-point Likert scale from 1 (never) to 5 (always). The second section has four questions measuring the mother’s overall attention or distraction during interactions with the infant, scored on a scale from 1 to 5. Here, a score of 1 indicates no distraction, while a score of 5 represents complete distraction. One question in this section, which assesses attention to the infant during feeding and caregiving, is reverse-scored; in this case, a lower score reflects higher attention. The validity and reliability of the MDQ were assessed by Ventura et al. [13], with Cronbach’s alpha of 0.86 for technological activities and 0.95 for internal consistency. In Iran, the MDQ was validated by Arezi et al. in Persian language, who reported an internal reliability coefficient of 0.75 using Cronbach’s alpha and a validity score of 0.67 [9].

### 2.6 Statistical analysis

Data were analyzed using SPSS software, version 16. Descriptive statistics were employed to assess maternal distraction levels based on scale scores. The Kolmogorov–Smirnov test confirmed a normal distribution for the total attention/distraction scale scores. Comparisons of total scores across demographic variables were conducted using independent *t*-tests and ANOVA, with a 95% confidence level. When the ANOVA results were statistically significant, pairwise comparisons were performed using the Bonferroni correction method.


**Informed consent:** Informed consent was obtained from all individual participants included in the study.
**Ethical approval:** The study protocol was accepted by the ethical committee of Hamadan University of Medical Sciences with code IR.UMSHA.REC.1403.085. We performed this study in accordance with the Declaration of Helsinki.

## 3 Results

All mothers (280 mothers) participated in this study. The age range of mothers was between 18 and 44 years. Table 1 shows the characteristics of the mothers included in the study. The mean age of mothers and babies was 30.06 ± 6.10 years and 2.74 ± 1.54 months, respectively.

**Table 1:** Characteristics of the mothers included in the study (*n* = 280)

Variables	*N* (%)
**Baby’s gender**	
Male	146 (52.1)
Female	134 (47.9)
Total	280 (100)
**Mother’s job**	
Employed	28 (10.0)
Housekeeper	252 (90.0)
Total	280 (100)
**Economic status**	
Good	77 (27.5)
Average	157 (56.1)
Weak	46 (16.5)
Total	280 (100)
**Number of children**	
1	113 (40.4)
2	124 (44.3)
3>	43 (15.4)
Total	280 (100)
**Mother’s education**	
<Diploma	68 (24.3)
Diploma	128 (45.4)
Academic	84 (30.3)
Total	280 (100)
	Mean (SD)
**Mother’s age (years)**	30.06 ± 6.10
**Baby’s age (months)**	2.74 ± 1.54

Table 2 shows that 61.08% of mothers had watched TV at least once while breastfeeding. Distraction during breastfeeding was reported by 40.36% of mothers due to computer use, 62.14% due to talking on the phone, 75% due to mobile phone use, and 6.78% due to reading a book. The highest distraction during breastfeeding was associated with mobile phone use, while the lowest was linked to reading. Additionally, findings show that 75.35% of mothers had watched TV at least once while providing baby care. Distraction during baby care was reported by 55.71% of mothers due to computer use, 71.07% due to talking on the phone, 79.64% due to mobile phone use, and 12.86% due to reading a book. Mobile phone use was the highest distraction during breastfeeding and baby care, while the lowest was reading a book.

**Table 2:** The frequency of distractions for mothers while breastfeeding their infants, or baby care while using a mobile phone, computer, or tablet (*n* = 280)

Breastfeeding	Never, *N* (%)	Rarely, *N* (%)	Sometimes, *N* (%)	Most of the time, *N* (%)	Always, *N* (%)
Watch TV (e.g., movies, shows, or videos)	109 (38.93)	53 (18.93)	92 (32.86)	21 (7.50)	5 (1.79)
Using a computer (e.g., check, surf the internet, work)	167 (59.64)	30 (10.71)	70 (25)	12 (4.29)	1 (0.36)
Talk on the phone	106 (37.86)	60 (21.43)	86 (30.71)	23 (8.21)	5 (1.79)
Text or use apps on a mobile device or tablet	70 (25)	65 (23.21)	132 (47.14)	12 (4.29)	1 (0.36)
Read a book, magazine, or newspaper (not on a mobile device or tablet	261 (93.21)	4 (1.43)	13 (4.64)	2 (0.71)	—
**Baby care**					
Watch TV (e.g., movies, shows, or videos)	69 (24.64)	90 (32.14)	96 (34.29)	16 (5.71)	9 (3.21)
Using a computer (e.g., check, surf the internet, work)	124 (44.29)	65 (23.21)	82 (29.29)	6 (2.14)	3 (1.07)
Talk on the phone	81 (28.93)	69 (24.64)	94 (33.57)	29 (10.36)	7 (2.50)
Text or use apps on a mobile device or tablet	57 (20.36)	72 (25.71)	141 (50.36)	6 (2.14)	4 (1.43)
Read a book, magazine, or newspaper (not on a mobile device or tablet	244 (87.14)	22 (7.86)	12 (4.29)	2 (0.71)	—

The findings in Table 3 indicate that the mean distraction of mothers from their babies during breastfeeding and caregiving was significantly associated with the baby’s age and the number of children. The results of the *post hoc* analysis showed that the pairwise comparison of the mean distraction scores during breastfeeding (*p*-value =0.007) and during child care (*p*-value <0.001) between women with three or more children and women with one child has a statistically significant difference. Additionally, the mean distraction score during child care between women with three or more children and women with two children also shows a statistically significant difference (*p*-value =0.04). Furthermore, the mean attention score between women with three or more children and women with two and one child also has a statistically significant difference (*p*-value < 0.05). Also, the level of distraction during breastfeeding and baby care among mothers of 6-month-old babies increased significantly. The pairwise comparison of the mean distraction scores during breastfeeding and child care was statistically significant only between women with 2- and 3-month-old children (*p*-value <0.001).

**Table 3:** The frequency of attention and distraction during breastfeeding and childcare based on background variables (*n* = 280)

Variables		Breastfeeding				Baby care			
	*N* = 280	Distraction	*p*	Attention	*p*	Distraction	*p*	Attention	*p*
		Mean (SD)		Mean (SD)		Mean (SD)		Mean (SD)	
**Mother’s age (years)**									
<25	71	1.59 (0.83)	0.25	4.33 (0.92)	0.21	1.81 (0.83)	0.07	4.18 (0.88)	0.86
25–30	68	1.79 (1.07)		4.16 (0.95)		1.87 (0.96)		4.14 (0.79)	
30–35	83	1.77 (0.91)		4.16 (0.92)		1.78 (0.92)		4.12 (0.91)	
>35	58	1.93 (1.02)		3.98 (0.99)		2.17 (0.94)		4.05 (0.96)	
**Baby’s age (months)**									
1	71	1.78 (0.97)	<0.001	4.21 (0.86)	0.09	1.92 (0.89)	<0.001	4.04 (0.92)	0.57
2	75	2.08 (1.03)		3.92 (1.06)		2.25 (1.02)		4.05 (0.83)	
3	60	1.40 (0.69)		4.36 (0.92)		1.65 (0.77)		4.23 (0.85)	
4	22	1.59 (0.66)		4.04 (0.95)		1.68 (0.56)		4.09 (0.97)	
5	33	1.57 (0.90)		4.33 (0.92)		1.45 (0.66)		4.33 (0.98)	
6	19	2.10 (1.28)		4.26 (0.80)		2.10 (1.15)		4.10 (0.81)	
**Baby’s gender**									
Male	146	1.79 (1.01)	0.58	4.14 (1.01)	0.61	1.86 (0.90)	0.66	4.13 (0.90)	0.86
Female	134	1.73 (0.90)		4.20 (0.87)		1.92 (0.94)		4.11 (0.86)	
**Mother’s job**									
Employed	28	1.53 (0.69)	0.18	4.32 (0.82)	0.37	1.71 (0.76)	0.89	4.25 (0.75)	0.44
Housekeeper	252	1.79 (0.98)		4.15 (0.96)		1.91 (0.93)		4.11 (0.90)	
**Mother’s education**									
<Diploma	68	1.89 (1.00)	0.23	3.91 (1.07)	0.02	2.05 (0.99)	0.04	4.10 (0.88)	0.74
Diploma	128	1.78 (0.94)		4.28 (0.83)		1.92 (0.90)		4.10 (0.86)	
Academic	84	1.63 (0.94)		4.20 (0.98)		1.70 (0.86)		4.19 (0.92)	
**Husband’s age (years)**									
<30	67	1.82 (1.02)	0.06	4.29 (0.97)	0.38	1.92 (0.96)	0.36	4.19 (0.87)	0.43
30–35	92	1.57 (0.87)		4.17 (0.95)		1.78 (0.82)		4.18 (0.75)	
>35	121	1.87 (0.97)		4.09 (0.93)		1.95 (0.98)		4.04 (0.98)	
**Husband’s education**									
<Diploma	88	1.84 (0.99)	0.55	4.01 (1.05)	0.16	1.94 (0.96)	0.82	4.08 (0.92)	0.81
Diploma	107	1.69 (0.87)		4.24 (0.90)		1.86 (0.84)		4.15 (0.87)	
Academic	85	1.77 (1.04)		4.24 (0.87)		1.87 (0.97)		4.14 (0.87)	
**Husband’s job**									
Employee	62	1.66 (0.86)	0.20	4.29 (0.91)	0.38	1.87 (0.84)	0.30	4.17 (0.87)	0.88
Free	178	1.74 (0.98)		4.16 (0.97)		1.85 (0.93)		4.11 (0.87)	
Worker	40	2.00 (0.98)		4.02 (0.89)		2.10 (0.95)		4.12 (0.96)	
**Economic status**									
Good	77	1.72 (0.96)	0.04	4.22 (0.95)	0.41	1.80 (0.91)	0.25	4.10 (0.94)	0.58
Average	158	1.69 (0.91)		4.19 (0.94)		1.88 (0.90)		4.17 (0.86)	
Weak	45	2.08 (1.06)		4.00 (0.97)		2.08 (0.97)		4.02 (0.89)	
**Number of children**									
1	113	1.62 (0.90)	0.01	4.27 (0.97)	0.01	1.71 (0.83)	0.001	4.23 (0.76)	0.12
2	124	1.76 (0.98)		4.20 (0.87)		1.91 (0.95)		4.11 (0.96)	
>3	43	2.14 (0.96)		3.79 (1.01)		2.30 (0.94)		3.90 (0.92)	
**Delivery type**									
NVD	174	1.77 (0.97)	0.80	4.12 (0.99)	0.31	1.92 (0.88)	0.45	4.13 (0.88)	0.93
C/S	106	1.74 (0.94)		4.24 (0.88)		1.83 (0.98)		4.12 (0.89)	

The pairwise comparison of the mean attention scores during breastfeeding between women with education below a diploma and those with a diploma had a statistically significant difference (*p*-value =0.02). Additionally, the mean distraction score during child care between women with a university education and those with education below a diploma also showed a statistically significant difference (*p*-value =0.04).

Maternal distraction during breastfeeding was significantly associated with economic status (*p* = 0.04). The pairwise comparison of the mean distraction scores between women with an average economic status and women with a weak economic status showed a statistically significant difference (*p*-value = 0.04) (Table 3).

No statistically significant differences in maternal distraction or attention during breastfeeding and caregiving were observed based on maternal age, baby’s gender, mother’s occupation, husband’s occupation, husband’s age, husband’s education, or delivery type (*p* > 0.05) (Table 3).

## 4 Discussion

The results of this study indicate that various factors influence maternal distraction during breastfeeding and childcare. Mobile phone usage was identified as one of the most common causes of maternal distraction. The present study indicates that distraction between women with an average economic status was lower compared with women with a weak economic status. Distraction during child care between women with university education was lower compared with education below a diploma. In addition, attention during breastfeeding in women with education below a diploma was lower compared with those with a diploma. Distraction during breastfeeding and during child care between women with three or more children was higher compared with women with one child. Additionally, Distraction during child care between women with three or more children was higher compared with women with two children. Furthermore, attention between women with three or more children was lower compared with women with two and one child. The findings also revealed that the level of maternal distraction increased in 6-month-old infants during breastfeeding and childcare. The findings of the present study align with the results of Tang et al. [14], who reported that mothers with lower educational levels (high school diploma or less) experienced greater distraction during caregiving and decreased attention to the infant during breastfeeding. Similarly, the study by Li et al. emphasized that education and awareness are key factors in managing focus and reducing distractions during childcare [15]. However, Seifert et al. reported that the use of technology can also be beneficial [7]. Therefore, it can be suggested that if smartphones are used appropriately for example to access breastfeeding education and related programs, they may serve as valuable tools rather than sources of distraction.

The present study indicates that distraction between women with an average economic status was lower compared with women with a weak economic status. This aligns with the findings of Nazari et al. [16], which highlighted the relationship between economic pressures and decreased focus during infant care.

The study by Arezi et al. also demonstrated that older mothers experienced less distraction during childcare [9]. However, no significant relationship was observed in our study. This discrepancy may be related to the age of the children. In our study, the sample population was limited to mothers of exclusively breastfed infants under 6 months old who require more maternal attention. In contrast, the recent study included children up to two years of age as its target population.

The study by Arezi et al. found a high level of distraction due to mobile phone use during critical childcare and breastfeeding periods [9], which aligns with the present study’s findings. The research by Golen et al. [12] emphasizes that distraction in child care is primarily related to excessive use of technology. These findings are particularly significant in understanding the impact of technology on maternal distraction and the quality of parent–child interactions. This suggests that more mindful and limited use of electronic devices should be encouraged to reduce distraction.

Our study is consistent with similar research, including the work by Muppalla et al., which demonstrated that excessive use of digital technologies can negatively affect the quality of parent–infant interactions [17]. While some studies, aligned with our research, emphasize that technology can act as a source of distraction and cause negative interference in breastfeeding and childcare, other studies, such as Seifert and Cotten indicate that when used properly, technology can serve as a supportive tool in mother-infant interactions and improve communication and necessary support during this sensitive period [18].

The data suggest that mobile phone use is particularly prevalent among mothers during both breastfeeding and childcare and is significantly associated with distraction. However, there is a risk of conflating correlation with causation. For instance, although mothers with more children reported higher levels of distraction, it is unclear whether this is primarily due to mobile device use, increased overall workload, or other stressors. Therefore, the findings indicate associations, but they do not provide evidence of a direct causal effect of device use.

### 4.1 Limitation

There are some limitations in this study: (a) this study has a cross-sectional design, which can identify associations but cannot establish causality. Future research should include longitudinal or interventional studies to better understand how mothers’ mobile phone use may change over time and whether it genuinely influences breastfeeding or childcare outcomes. (b) The MDQ is a self-reported questionnaire completed by mothers. As a result, social desirability bias may lead participants to under-report phone use or over-report socially desirable behaviors. (c) Focusing on a specific geographical area (Hamadan) and considering only the exclusive breastfeeding period may limit the generalizability of the findings to other communities. (d) The lack of in-depth qualitative exploration of the causes of distraction overlooks the issue’s important cognitive and emotional aspects. (e) Maternal mental health factors, stress levels, or social supports can also affect a mother’s distraction and attention during breastfeeding and childcare, which were not addressed in this study. These limitations suggest that further studies with cohort and qualitative approaches require a better and more comprehensive understanding of the factors influencing maternal distraction.

### 4.2 Practical implications

Consequently, this study clarifies the factors related to maternal distraction during breastfeeding and highlights the interplay between economic status, education, and infant developmental stages. Addressing these distractions through targeted educational interventions could improve mother-infant interactions and yield better outcomes for both mothers and children. These findings also illuminate healthcare approaches and emphasize intervention strategies that enhance maternal focus in critical caregiving situations, ultimately strengthening the bond and improving the child’s developmental trajectory.

## 5 Conclusion

The present study indicates that distraction between women with an average economic status was lower compared with women with a weak economic status. Distraction during child care between women with university education was lower compared with education below a diploma. In addition, attention during breastfeeding in women with education below a diploma was lower compared with those with a diploma. Distraction during breastfeeding and during child care between women with three or more children was higher in compared with women with one child. Additionally, distraction during child care between women with three or more children was higher compared with women with two children. Furthermore, attention between women with three or more children was lower compared with women with two and one child.

## Abbreviation

MDQ: Maternal Distraction Questionnaire
